# Do Subliminal Fearful Facial Expressions Capture Attention?

**DOI:** 10.3389/fpsyg.2022.840746

**Published:** 2022-04-12

**Authors:** Diane Baier, Marleen Kempkes, Thomas Ditye, Ulrich Ansorge

**Affiliations:** ^1^Faculty of Psychology, University of Vienna, Vienna, Austria; ^2^Acoustics Research Institute, Austrian Academy of Sciences, Vienna, Austria; ^3^Faculty of Psychology, Sigmund Freud University, Vienna, Austria; ^4^Cognitive Science Hub, University of Vienna, Vienna, Austria

**Keywords:** subliminal, facial expression, emotion, attention, ERP

## Abstract

In two experiments, we tested whether fearful facial expressions capture attention in an awareness-independent fashion. In Experiment 1, participants searched for a visible neutral face presented at one of two positions. Prior to the target, a backward-masked and, thus, invisible emotional (fearful/disgusted) or neutral face was presented as a cue, either at target position or away from the target position. If negative emotional faces capture attention in a stimulus-driven way, we would have expected a cueing effect: better performance where fearful or disgusted facial cues were presented at target position than away from the target. However, no evidence of capture of attention was found, neither in behavior (response times or error rates), nor in event-related lateralizations (N2pc). In Experiment 2, we went one step further and used fearful faces as visible targets, too. Thereby, we sought to boost awareness-independent capture of attention by fearful faces. However, still, we found no significant attention-capture effect. Our results show that fearful facial expressions do not capture attention in an awareness-independent way. Results are discussed in light of existing theories.

## Introduction

Past research has shown that visual stimuli capture attention based on factors such as task relevance (Folk et al., 1992; Büsel et al., 2020), visual salience (Nothdurft, 1993; Itti et al., 1998; Wang and Theeuwes, 2020), or prior individual experience with these stimuli (Awh et al., 2012). It is more contested, however, if some visual stimuli can capture attention based on their general phylogenetic relevance (cf. Öhman, 1993; Öhman et al., 2001), a prime example of which is the capture and holding of attention by specific emotional facial expressions (e.g., Eastwood et al., 2001; Fox et al., 2001; Vuilleumier, 2005; Bannerman et al., 2010; Van Hooff et al., 2013). Critically, several studies suggested that fearful faces could capture attention even if task-irrelevant (Bannerman et al., 2010) and that this effect could not be ascribed to visual salience alone (Khalid et al., 2017). Khalid et al. (2017) carefully controlled for mere influences of salience by presenting faces as cues either in their cardinal orientation or upside-down. The authors found that task-irrelevant fearful faces only captured attention if presented in their cardinal orientation, indicating that emotion recognition was relevant for the capture of attention (cf. Huynh and Balas, 2014). Salience, in contrast, was insufficient to explain the capture of attention, as salience was the same for cardinal and inverted orientations. The observation of salience independence is critical, as otherwise the visual salience of a particular facial expression itself could capture attention merely through basic visual and emotion-unspecific characteristics (cf. Morris et al., 2002; Horstmann and Bauland, 2006).

Apart from task-irrelevant capture by liminal emotional faces, researchers have looked into electrophysiological data during task-relevant perception of subliminally presented faces at central locations. Findings for early components were indecisive, with some studies showing early differential processing of fearful compared to neutral facial expressions (Kiss and Eimer, 2008; Pegna et al., 2008; Smith, 2012; Zhang et al., 2012, 2017) while others do not (Liddell et al., 2004; Walentowska and Wronka, 2012). In addition, subliminal processing was only verified on a trial-by-trial basis in some studies (Pegna et al., 2008; Smith, 2012; Zhang et al., 2012), of which two found an increased N170 for subliminal fearful faces (Pegna et al., 2008; Smith, 2012).

Here, we went one step further and tested the important question of whether the known capture effects of task-irrelevant fearful faces observed by Khalid et al. (2017) extend to subliminally presented emotional faces, as the general human ability to differentially process subliminal emotional expressions would suggest (cf. Kiss and Eimer, 2008; Pegna et al., 2008). The question is important considering alternative theories arguing for prominent roles of awareness independence vs. awareness dependence in human social and emotional processing abilities. On the one hand, some theories argue that the rapid processing of emotional facial expressions provided a phylogenetic benefit pre-dating consciousness and, thus, the corresponding capture of attention by emotionally significant facial displays could be awareness-independent (Morris et al., 1998; De Gelder et al., 1999; Öhman, 2002; Dolan and Vuilleumier, 2003; Tamietto and De Gelder, 2010). On the other hand, some theories emphasized the role of awareness or consciousness in some social and emotional skills (Frith and Frith, 2007; Frith, 2008). Authors argued for a role of awareness at least for forms of more deliberate social processing, taking intentions into account. Likewise, emotions themselves have been described as the result of both, more automatic and more reflective processes (see, for example, Weiner, 1986). The higher cognitive processes are involved when we have to make sense of the behavior of others. Now, it might seem that the processes triggered by stimuli, such as specific emotional faces, could be relatively simple, sparing the more complex forms of emotional and social processing that depend on awareness. However, in this case it is also important to concede that manipulations of awareness and visibility carry the risk of sacrificing much of this processing efficiency (cf. Hedger et al., 2016).

In the present study, we therefore tested whether masked (subliminal) fearful faces capture attention. In contrast to the classic dot-probe task, where emotional faces are used as cues and an onset stimulus is shown as target (e.g., a dot as location target or an orientated shape as discrimination target; see Mogg and Bradley, 1999; Holmes et al., 2005; Cooper and Langton, 2006; Brosch et al., 2008; Puls and Rothermund, 2018), we used two stimuli in the target display, instead of only one onset target (cf. Wirth and Wentura, 2018, 2019). We applied this variation of the dot-probe paradigm, a visual search task for a predefined face target presented unforeseeably at one of two positions, following Khalid et al. (2017). The visual search for target faces has some decisive advantages compared to the classic dot-probe task with onset targets. In particular, the task of searching for a target face allowed us to vary the task relevance of the masked (subliminal) fearful faces, as we will explain in more detail below.

To start with, prior to the face target, we presented a fearful face as a cue either at the position of the incumbent target (valid condition) or at the alternative position (invalid condition). Although the cues were on average not predictive of the target position, in line with prior studies with supraliminal fearful face cues (cf. Khalid et al., 2017), we expected a validity effect if the subliminal fearful face cues captured attention, too: faster responses to validly cued targets than to invalidly cued targets. However, we were aware that in the small cue-target interval more complex processes could occur than a mere capture of attention and dwelling of attention at the cued position lasting until (or even after) the target is presented. For example, it is possible that cues initially capture attention, but that attention is then deallocated from the cue and shifted back to a neutral position prior to the target (Theeuwes et al., 2000). Likewise, it is possible that the fearful face cue captures attention but that target search is then so easy that one would literally not see any evidence of attention dwelling at the cued position, once target displays have commenced (Gaspelin et al., 2016). Therefore, for our test of attention capture by subliminal fearful face cues, we used a more exhaustive measure than mere reaction time effects. We used the electroencephalogram (EEG) to test for attention-elicited event-related lateralizations (ERLs; Luck and Hillyard, 1994; Luck, 2012). For example, Eimer and Kiss (2007) found attention capture by irrelevant fearful faces in the form of an N2pc, a stronger negativity at about 200–250 ms following stimulus onset at contra-compared to ipsilateral posterior scalp sites that was reflective of an attention shift to stimuli presented to the left or to the right. In addition, such N2pc effects can also be elicited by subliminal stimuli (e.g., color cues; Ansorge et al., 2009). To find out whether a subliminally presented fearful face cue captures attention, we conducted two cueing experiments (Posner, 1980) and used EEG to look for cue-elicited ERLs. In Experiment 1, we used task-irrelevant fearful and disgusted faces as cues, each presented with a task-relevant neutral face at the opposite position. In Experiment 1, we would have expected validity effects by masked fearful faces and maybe by masked disgusted faces if these subliminal cues captured attention in a similar way as their supraliminal counterparts used in Khalid et al. (2017). These authors found orientation-specific cuing effects for fearful faces but orientation-independent cueing effects for disgusted faces, showing that capture by fearful faces is emotion-specific. In the present Experiment 1, participants had to search for neutral face targets, meaning that the fearful face cues were task-irrelevant. However, we admit that we thereby created a relatively conservative test of the fearful face cues’ potential to capture attention, as the task-relevant neutral face stimulus on the opposite side of the fearful face cue would have matched the top-down attentional control settings (or search criterion) of the participants for the targets. Thus, if neutral face stimuli presented opposite to the fearful face cues would capture attention in a top-down contingent way, this would counteract the capture of attention by the task-irrelevant face cue. Therefore, in Experiment 2, we made the negative facial expression of fear task-relevant, in order to boost attention-capture effects by subliminally presented fearful face cues. In both experiments, face cues were presented in upright or in inverted orientation. As in Khalid et al. (2017), this was done to rule out salience as a potential explanation of any cueing effect: If a capture effect was due to salience, we expected to find it both in upright and inverted conditions. However, a capture effect that was due to the specific emotional expression of the cues was expected to only show in the upright orientation, but not in the inverted orientation.

## Materials and Methods

### Participants

Thirteen participants were tested in Experiment 1 (*M*_age_ = 24.31 years, SD_age_ = 2.36 years) and 16 in Experiment 2 (*M*_age_ = 22.28 years, SD_age_ = 4.41 years). They signed an informed consent form after being informed about their tasks and rights. No approval from the ethics committee of the University of Vienna was necessary (according to the Austrian Universities Act of 2002), as the study did neither offer incidental findings of clinical or diagnostic relevance, nor threaten the participants’ physical or psychological integrity, their right to privacy, other subjective rights, nor other prevailing interests. The duration of the computer experiment was 40 min. Participants sat in a quiet, dimly, and indirectly lit room. Their distance to the screen (60 cm) was kept constant by a chinrest. Including breaks and preparation for electrophysiological data collection, they spent between 2.5 and 3 h in the lab.

### Stimuli, Design, Task, and Procedure

**Figure 1:**
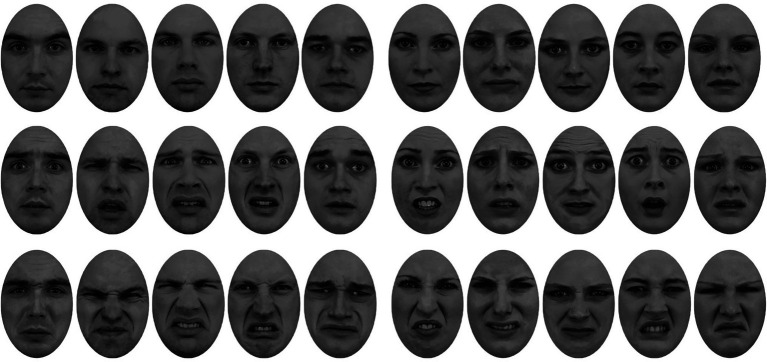
Face stimuli used in Experiments 1 and 2 (Lundqvist et al., 1998; Khalid et al., 2017): neutral faces (top row), fearful faces (middle row), and disgusted faces (bottom row). Males are on the left, females on the right.

The stimuli for this study were a selection from the Karolinska Directed Emotional Faces (KDEF) database (Lundqvist et al., 1998; see also Khalid et al., 2017). The grayscale images included five female and five male faces with fearful, disgusted, and neutral expressions. All images were equated for luminance, contrast, and spectral power and cropped behind a white over layer, to ensure that only the face features were visible (see Figure 1).

In our experiments, the images were presented with an eccentricity of 9.2° on the left and right side of the display and subtended a visual angle of 11.2° vertically and 7.5° horizontally. Face identity changed in every trial, face gender was randomized, and potential repetition was limited to five succeeding trials. A fixation cross was displayed in the middle of the screen throughout the trial and preceded stimulus presentation for 1 s. In each trial, we then presented a face cue, defined as bearing an irrelevant negative facial expression (disgusted or fearful; Experiment 1) or a target-search relevant fearful expression (Experiment 2) together with a neutral face as distractor. Each cueing display, thus, consisted of two faces, side by side, one on the left and one on the right: one neutral face distractor and one negative emotional (fearful/disgusted) face cue in Experiment 1; or one fearful face cue and one neutral or disgusted face distractor in Experiment 2. The face cues were either presented upright or inverted (see Table 1 for the experimental designs of Experiments 1 and 2).

**Table 1:** Experimental design of Experiments 1 and 2.

	Cueing display			Target display
	Orientation	Facial expressions	Validity	Facial expressions
Experiment 1	Upright	Neutral & fearful	Valid	Disgusted & neutral
			Invalid	Neutral & disgusted
		Neutral & disgusted	Valid	Disgusted & neutral
			Invalid	Neutral & disgusted
	Inverted	Neutral & fearful	Valid	Disgusted & neutral
			Invalid	Neutral & disgusted
		Neutral & disgusted	Valid	Disgusted & neutral
			Invalid	Neutral & disgusted
Experiment 2	Upright	Fearful & neutral	Valid	Fearful & neutral
			Invalid	Neutral & fearful
		Fearful & disgusted	Valid	Fearful & neutral
			Invalid	Neutral & fearful
	Inverted	Fearful & neutral	Valid	Fearful & neutral
			Invalid	Neutral & fearful
		Fearful & disgusted	Valid	Fearful & neutral
			Invalid	Neutral & fearful

Cue orientation alternated block-wise; all other conditions were randomized throughout blocks.

**Figure 2:**
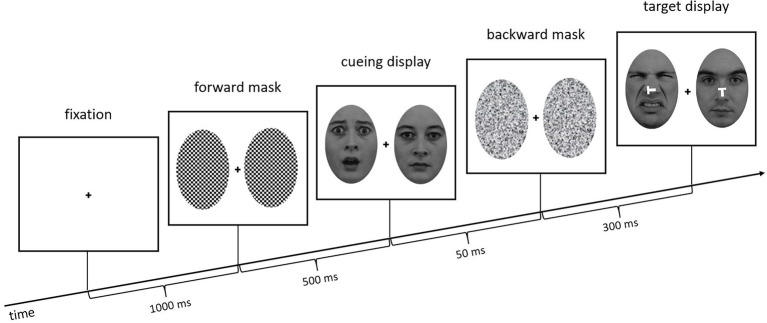
Exemplary trial sequence of Experiment 1. The target face (neutral face) is at the right side, the distractor (disgusted face) on the left. In the cueing display, the neutral face is on the right side, and the fearful face cue is at the left (distractor position in the target display, hence, an invalid trial). Stimuli are not drawn to scale.

To present the face cues subliminally, they were only shown for 50 ms and additionally sandwiched between forward masks (checkerboard structure, 500 ms) and backward masks (scrambled faces, 300 ms; cf. Kahneman, 1968; Esteves and Öhman, 1993). The task was to find the target face (Experiment 1: neutral face; Experiment 2: fearful face) next to a distractor face (Experiment 1: disgusted face; Experiment 2: neutral face) and press a key corresponding to the orientation of the small white *T* in the center of the target face (Keys #2, #4, #6, and #8 on the keypad of a standard keyboard). The target display was presented for 200 ms (followed by a blank screen until a response was given) and the *T*s, which appeared 20 ms after the target faces, were either upright, inverted, flipped to the left, or flipped to the right (see Figure 2), requiring an orientation-compatible button press starting from the central home key (Key #5) to the top, the bottom, to the left, or to the right, respectively. Using four different letter orientations and responses meant that we could create incongruent relations between the two letters in the target display—the one at target position and the one at distractor position—without letters at the distractor position ever specifying the required responses. (For comparison, with only two orientations and responses, using only incongruent letter orientations would have meant that the letter at distractor position would have informed about the required response, too. This was to be prevented, as participants would otherwise not have to search and could simply wait for the stimulus at a single pre-selected location and respond to it depending on a combination of facial expression and letter orientation).

Before starting with the main experiment (eight blocks à 60 trials), participants completed 10 practice trials. To verify the subliminal presentation of the face cues, we included two dual-task blocks (à 60 trials) after the main-task blocks. Here, in addition to the main task, the question on which side of the cueing display the neutral face was presented in Experiment 1 or on which side the fearful face was shown in Experiment 2, had to be answered by keypress.

### Data Collection and Preprocessing

The monitor used for visual presentation was a 19” VGA monitor (1,024 × 786 pixels; refresh rate: 59 Hz). The experiments were programmed and conducted with the Psychophysics Toolbox (Brainard, 1997) in MATLAB (2013, The MathWorks, Natick, MA). We collected electrophysiological data with 64 active electrodes (actiCAP, Brain Products GmbH, Gilching, Germany) in a 10/10 system cap (EASYCAP GmbH, Herrsching, Germany) at a sampling rate of 1,000 Hz with a neuroConn amplifier (neuroConn GmbH, Ilmenau, Germany). The ground electrode (AFZ) was used as online reference during recording. For offline re-referencing, we calculated the average of both mastoids. We applied a 40 Hz low-pass finite impulse response filter (cutoff 45 Hz; transition band width 10 Hz). The horizontal electrooculogram (HEOG) was attained by the difference between the two electrodes positioned at the outer canthi. Trial rejection was done separately for every channel. Here, trials with very low activity (less than 0.5 μV difference between subsequent samples within a period of 500 ms), very high signal changes (more than 50 μV/ms), values exceeding 80 μV, HEOG exceeding ±30 μV, and vertical eye movements or blinks (Fp1/Fp2 ± 60 μV) were removed (19.3% in Exp. 1, 26.18% in Exp. 2), as well as trials with wrong behavioral responses. EEG data was processed in MATLAB using the EEGLAB toolbox (Delorme and Makeig, 2004) with the ERPLAB extension (Lopez-Calderon and Luck, 2014). For analyses of event-related potentials (ERPs), we extracted mean amplitudes between two fixed latencies and analyzed them in *R* (R Core Team, 2017) using the packages *apa* (Gromer, 2017) and *ez* (Lawrence, 2016). The respective time windows were chosen based on the literature and refined using visual inspection of collapsed waveforms (collapsed-localizers method; Luck and Gaspelin, 2017).

## Experiment 1

In Experiment 1, we wanted to find out whether task-irrelevant, subliminally presented fearful facial expressions automatically capture attention and to what extent this effect could be specific to fear vs. disgust. To note, fear has an arguably higher potential to capture attention in an emotion-specific (orientation-dependent) way (cf. Khalid et al., 2017). Participants were instructed to search for a neutral face. Prior to the target faces, we presented participants both neutral cue distractors and negative emotional (fearful or disgusted) face cues, which were oriented either upright or inverted.

### Hypotheses

If the task-irrelevant fearful faces captured attention in an awareness-independent fashion, we expected to find an N2pc component to the face cues in the cueing display, depending on the position of the fearful face. In addition, we expected to find cueing effects in the behavioral data: faster responses and fewer errors for valid trials (fearful cue at neutral target’s position) than invalid trials (fearful cue at opposite side of neutral target’s position). If the capture effect was fear-specific, we expected to see the validity effect in the conditions with fearful face cues only. However, if the effect is due to the negativity of the emotional valences, we expected to see the validity effect for both fear and disgust cues. In addition, if the effect was face- and, thus, emotion-specific, we expected to see more capture in upright than in inverted face-distractor conditions (cf. McKelvie, 1995; Khalid et al., 2017). Critically, if we found no such effects for any of the negative emotional face cues, but for the neutral face distractors (which are congruent with the search goal), we would conclude that task relevance overrides the evolutionary salience of negative facial expressions.

For the additional visibility or awareness test of the masked cues in the dual-task phase (identifying presentation side of neutral face cue), we expected a discrimination accuracy at chance level if cue faces were presented subliminally, as subliminal presentation should not allow for awareness and, hence, prevent correct classification of the masked facial expressions.

### Event-Related Potentials

**Figure 3:**
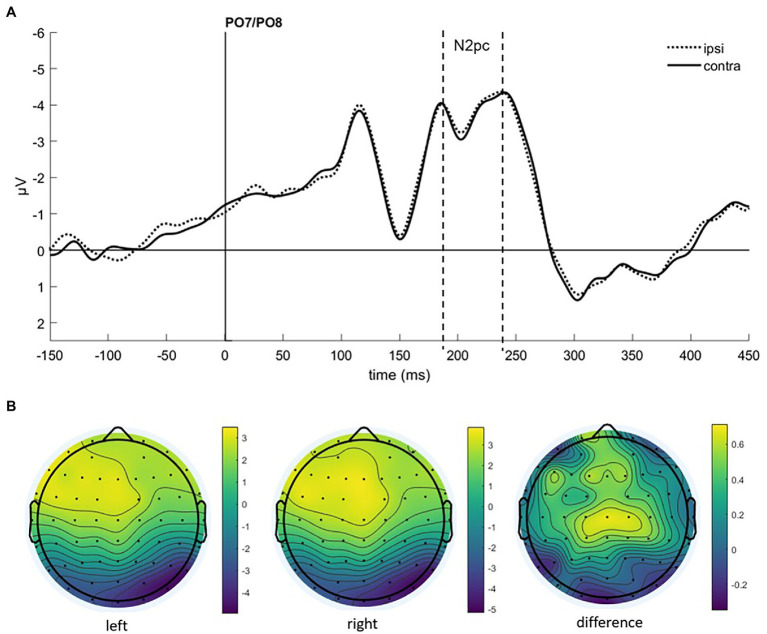
**(A)** Event-related potentials (ERPs) in Experiment 1 after cue onset collapsed over experimental conditions. Activity contralateral to negative emotional face cues is represented by a solid line, ipsilateral by a dotted line. **(B)** Scalp topographies for left/right cue presentation and the difference (in μV) averaged over 190–240 ms after cue onset (N2pc).

To find evidence of attention capture by fearful or negative emotional face cues in the electrophysiological data, we looked at the N2pc component. It usually occurs between 200 and 300 ms after stimulus onset at the electrode sites PO7 and PO8, with a more negative distribution at the electrode on the hemisphere contralateral to the presentation side of the attended stimulus (e.g., Luck and Hillyard, 1994; Eimer and Kiss, 2007; Jolicæur et al., 2008), also under masked conditions (Woodman and Luck, 2003; Ansorge et al., 2009). After visual inspection of collapsed data (see Figure 3), we set the time window for the N2pc to 190–240 ms.

**Figure 4:**
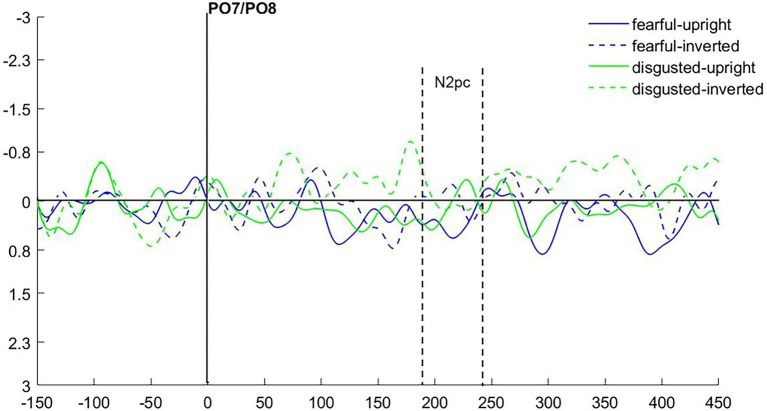
Differences in contra- minus ipsilateral event-related potentials (ERPs) in Experiment 1 after cue onset, depending on Negative Emotion and Orientation of the cue.

As we were interested in attention capture by the masked task-irrelevant negative face cues, the N2pc was defined based on the location of the fearful/disgusted face cues in the cueing display. One participant had to be excluded from the EEG analysis because of insufficient data quality. With the data of the remaining 12 participants, we ran an analysis of variance (ANOVA) with the mean amplitude values as dependent variables, and the independent variables Hemisphere (contra- or ipsilateral), Orientation (upright or inverted) and Negative Emotion of the cue (fearful or disgusted). We found no significant effects, all *F*s(1, 11) < 0.86, all *p*s > 0.373, all $\eta_{p}^{2}$ s < 0.07 (see Figure 4). In addition, we calculated the Bayes factor using JASP (JASP Team, 2019) for the same models included in the ANOVA. All respective Bayes factors for the N2pc (factor Hemisphere) showed substantial evidence for the H_0_ (no difference), all *BF*_10_ < 0.307, see Table 2.

**Table 2:** Bayes factors (BF_10_) for main effect and interaction models with mean amplitude differences (N2pc) of Experiments 1 and 2, as well as for the combined analysis of both experiments.

Model	BF_10_		
	Exp. 1	Exp. 2	Exp. 1 and 2
Hemisphere	0.245	0.207	0.206
Orientation	0.307	0.270	0.313
Emotion	0.274	0.824	n.a.
Hemisphere × Orientation	0.076	0.053	0.063
Hemisphere × Emotion	0.089	0.169	n.a.
Orientation × Emotion	0.067	0.220	n.a.
Hemisphere × Orientation × Emotion	0.021	0.044	n.a.

### Behavioral Results

**Figure 5:**
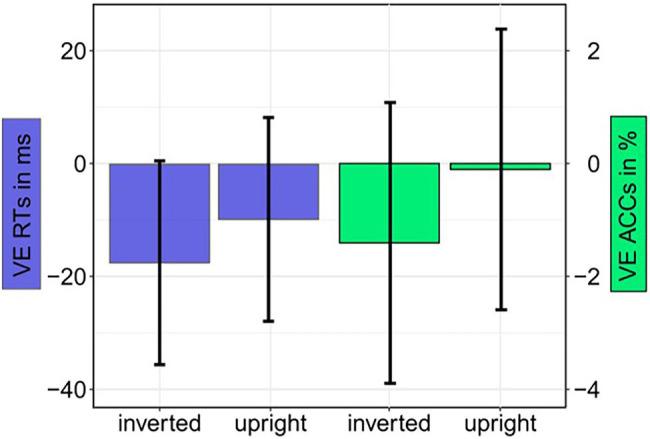
Experiment 1: Mean validity effect (VE) in reaction times (RTs; invalid minus valid; blue bars) and accuracies (ACCs; valid minus invalid; green bars) depending on Cue Orientation (upright; inverted). Error bars represent 95% confidence intervals.

For the main task, we calculated ANOVAs, with the independent variables Orientation (upright or inverted cues), Validity (emotional face in cueing display at same or different position as/than the neutral face in the target display), and Negative Emotion of the cue (fearful or disgusted face cue; to find possible differences between masked fearful and disgusted negative expressions). For the analysis of reaction times (RTs), only correct responses within two *SD*s from the median per person per condition were included (72.1%). There were no significant results, neither for the RTs, all *F*s(1, 12) < 1.43, all *p*s > 0.225, all $\eta_{p}^{2}$ s < 0.11, nor the accuracies (ACCs), all *F*s(1, 12) < 4.13, all *p*s > 0.065, all $\eta_{p}^{2}$ s < 0.26 (see Figure 5 and Table 3). The respective Bayes factors showed no evidence for the main effect of Validity (RT: *BF*_10_ = 0.475; ACCs: *BF*_10_ = 0.412) and evidence for the H_0_ (no difference) for all other models (all *BF*_10_ < 0.282; ACCs: all *BF*_10_ < 0.306; see Table 4 for all *BF*_10_s).

**Table 3:** Mean reaction times (RTs) in ms and accuracies (ACCs) in % of Experiments 1 and 2 depending on validity and cue orientation.

Validity	Orientation	Exp. 1		Exp. 2	
		RTs	ACCs	RTs	ACCs
Valid	Upright	769	76.3	693	78.0
	Inverted	784	74.8	691	77.6
Invalid	Upright	757	76.4	685	78.8
	Inverted	763	76.5	692	79.4

**Table 4:** Bayes factors (BF_10_) for main effect and interaction models with reaction times (RTs) and accuracies (ACCs) of Experiments 1 and 2, as well as for the combined analysis of both experiments.

Model	Exp. 1		Exp. 2		Exp. 1 and 2	
	RT	ACC	RT	ACC	RT	ACC
Validity	0.475	0.306	0.198	0.627	0.330	0.836
Orientation	0.282	0.250	0.194	0.185	0.243	0.210
Emotion	0.223	0.412	0.296	0.218	n.a.	n.a.
Validity × Orientation	0.134	0.077	0.040	0.119	0.080	0.187
Validity × Emotion	0.103	0.128	0.042	0.142	n.a.	n.a.
Orientation × Emotion	0.063	0.102	0.040	0.041	n.a.	n.a.
Validity × Orientation × Emotion	0.028	0.032	0.008	0.027	n.a.	n.a.

To verify subliminal presentation of the face cues, we analyzed cue-discrimination performance (in the dual-task phase only). In 49.7%, the position of the cue was reported correctly. This value does not differ significantly from chance performance (50%), *t*(12) = −0.31, *p* = 0.762, *d* = −0.09.

## Experiment 2

In Experiment 1, neither the task-irrelevant masked negative emotional cues nor the search-goal congruent, task-relevant emotionally neutral face cues were strong enough to elicit attention capture effects. As masked emotional and task-relevant faces were presented simultaneously in the cueing display, their respective capture effects might have cancelled each other out. To boost the effect of the subliminal negative expressions, in Experiment 2, we changed the task: Now, participants had to search for a fearful expression in the target display (and report the orientation of a letter *T* there). In this situation, the masked fearful cue face became task-relevant, as it shared its type of emotional expression with the target. As the fearful cue face was both task-relevant and (evolutionary) salient, its ability to capture attention should have been enhanced. By the same logic, the masked neutral face in the cueing display was now task-irrelevant. It was expected to lose the power it may have had to capture attention in a top-down contingent way in Experiment 1. All in all, the chances for capture of attention by the subliminal fearful cue faces relative to the neutral distractor faces was, thus, enhanced in Experiment 2 relative to that of the fearful face distractors in Experiment 1.

### Hypotheses

If subliminal fearful faces can capture attention, we expected to find an N2pc, as well as validity effects in behavioral data based on the position of the fearful cue faces, as those masked faces were now relevant, as they carried an expression shared by the targets. To note, each fearful face target in Experiment 2 was accompanied by a neutral face distractor, so that participants had to search for the target by its facial expression. However, as participants might have also based their search for the targets on the targets’ negative emotional expressions rather than on the particular emotional expression of fear, emotionally negative disgusted masked face distractors in the cueing displays might have captured attention in a top-down dependent way, too. In any case, the capture effects should have been weaker for the masked neutral face distractors. In line with our hypotheses for Experiment 1, these effects should only be present or at least be stronger for upright cues (if the effects are due to emotions rather than salience *per se*), and discrimination performance of masked faces in the dual-task phase should have been at chance level.

### Event-Related Potentials

**Figure 6:**
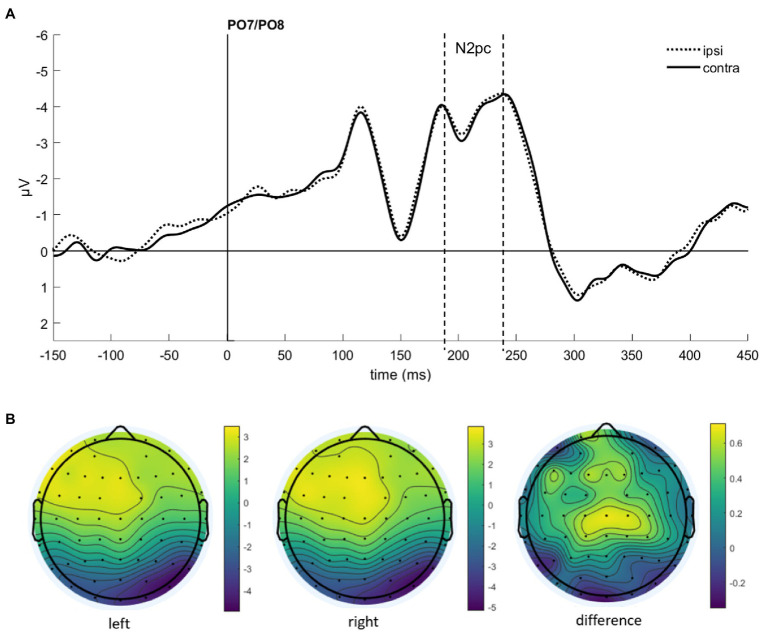
**(A)** Event-related potentials (ERPs) in Experiment 2 after cue onset collapsed over experimental conditions. Activity contralateral to the fearful emotional cue is represented by a solid line, ipsilateral by a dotted line. **(B)** Scalp topographies for left/right cue presentation and difference (in μV) averaged over 200–250 ms after cue onset (N2pc).

As in Experiment 1, we calculated the N2pc based on the location of the emotional (fearful and disgusted) masked cue faces. As the latencies were slightly shifted in Experiment 2 (see Figure 6 for ERPs collapsed over experimental conditions), we set the time window of our analysis to 200–250 ms after cue onset.

**Figure 7:**
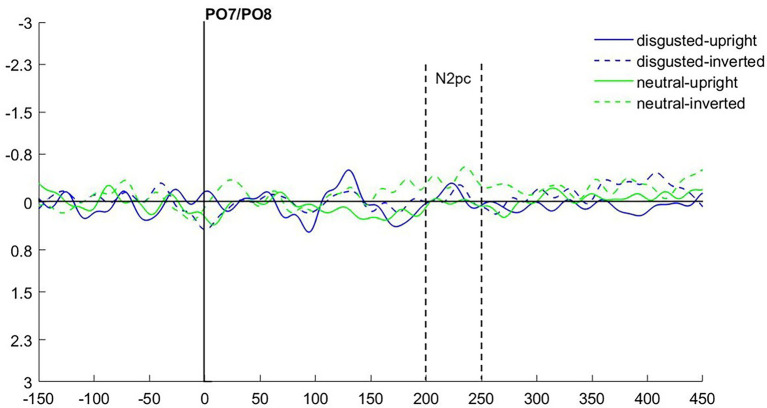
Differences in contra- minus ipsilateral event-related potentials (ERPs) in Experiment 2 after cue onset depending on cueing displays’ Distractor Emotion and Cue Orientation.

We ran an ANOVA with the mean amplitude values as dependent variable, and the independent variables Hemisphere (contra- or ipsilateral), Orientation (upright or inverted), and Distractor Emotion (disgusted or neutral distractor in the cueing display). We found no significant effects, all *F*s(1, 15) < 2.32, all *p*s > 0.149, all $\eta_{p}^{2}$ s < 0.13 (see Figure 7). The Bayes factor for the main effect of Negative Emotion contained no evidence, *BF*_10_ = 0.824, while all other *BF*_10_ < 0.270 yielded evidence for the H_0_ (no difference; see Table 2 for all Bayes factors).

### Behavioral Results

**Figure 8:**
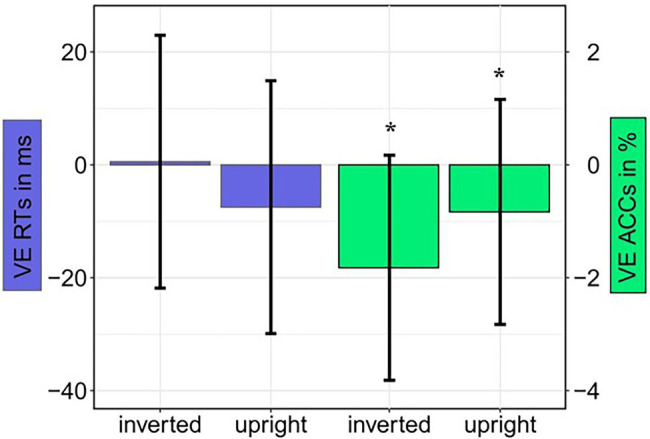
Experiment 2: Mean validity effect (VE) in reaction times (RTs; invalid minus valid; blue bars) and accuracies (ACCs; valid minus invalid; green bars) depending on Orientation (upright; inverted). Error bars represent 95% confidence intervals.

In line with the analysis in Experiment 1, we calculated ANOVAs, with the independent variables Orientation (upright or inverted cues), Validity (fearful face in cueing and target display at same position or different positions), and Distractor Emotion (neutral or disgusted distractor in the cueing display; in order to find possible differences between distraction by emotional and non-emotional distractors). Only correct responses within 2 SDs from the median per person per condition were included for analysis of the RTs (74.0%). In the ANOVA, nothing was significant, all *F*s(1, 15) < 2.66, all *p*s > 0.124, all $\eta_{p}^{2}$ s < 0.15. For the ACCs, only the main effect of validity was significant, *F*(1, 15) = 7.91, *p* = 0.013, $\eta_{p}^{2}$ = 0.35, and nothing else, all *F*s(1, 15) < 2.98, all *p*s > 0.105, all $\eta_{p}^{2}$ s < 0.17. Contrary to our expectations, participants gave more correct answers when the fearful cue was at the target display’s distractor position (79.1%), compared to target position (77.8%; see Figure 8 and Table 3). The Bayes factor for the same models that were included in the ANOVA yielded evidence for the H_0_ (no difference) in the RTs, all *BF*_10_ < 0.296. In the ACCs, the main effect of Validity showed no evidence: *BF*_10_ = 0.627, all other *BF*_10_ < 0.218 yielded evidence for the H_0_ (no difference; see Table 4 for all *BF*_10_s).

Analysis of cue-discrimination performance in the dual-task phase showed that participants were able to discriminate the position of the fearful cue with a mean probability of 52.9%, which is slightly better than chance performance, *t*(15) = 2.51, *p* = 0.024, *d* = 0.63.

## Power Calculations and Additional Analyses

To account for the relatively small sample size, we analyzed the N2pc component to the masked cue face pooled over Experiments 1 and 2. An ANOVA with the mean amplitude values from the separate analyses and the independent variables Hemisphere (contra- or ipsilateral) and Orientation (upright or inverted), again, revealed no significant effects, all *F*s(1, 27) < 1.73, all *p*s > 0.200, all $\eta_{p}^{2}$ s < 0.06. In addition, the BF_10_ = 0.206 (main effect N2pc) yields substantial evidence for the H_0_ (see Table 2 for a complete list of BF_10_s). A *post hoc* power analysis for the N2pc shows that with an *N* = 28 (*α* = 0.05, power = 0.8, one-sided), medium-sized effects (down to 0.48) could have been found.

In addition, we combined the behavioral data from Experiments 1 and 2 and did pooled analyses with RTs and ACCs and the independent variables Validity (valid/invalid) and Orientation (upright/inverted), as a *post hoc* power analysis (*α* = 0.05, power = 0.8) for the separate analyses revealed that only big effects (Exp. 1: *d* = 0.73; Exp. 2: *d* = 0.65) could have been found. In the pooled analysis (*N* = 29), still no effects were found for the RTs, all *F*s(1, 28) < 0.79, all *p*s > 0.383, all $\eta_{p}^{2}$ s < 0.03. For the ACCs, in line with the separate results from Exp. 2, only the main effect of Validity was significant, *F*(1, 28) = 7.92, *p* = 0.006, $\eta_{p}^{2}$ = 0.24 (valid: 76.8%; invalid 77.9%). See Table 4 for Bayes factors. Participants made more errors in valid than invalid cases. This might be caused by the relatively long interval between cue and target (350 ms), which was needed to analyze ERPs to the cue without distortion by the target screen. According to Klein (2000), this time interval might already subject processing at cued locations to some counteracting influences of inhibition of return.

## General Discussion

To examine attention capture by subliminally presented fearful facial expressions, we conducted two experiments and analyzed electrophysiological (lateralized ERPs) and behavioral data (RTs and ACCs). In Experiment 1, we found no evidence for attention capture by task-irrelevant, subliminally presented fearful facial expressions, neither in ERPs, nor in behavioral data. As the ipsilateral activity after cue onset was numerically even more negative than the contralateral (see Figure 3), which would represent an N2pc to the task-relevant neutral face, the attention-capture effects by the task-relevant neutral and task-irrelevant negative faces might have diminished each other. Therefore, in Experiment 2, we made the negative facial expression (fearful face) task-relevant. If in Experiment 1, both task-irrelevant fearful and task-relevant neutral faces competed for attention, in Experiment 2, the effects of task relevance and fearful emotion should add up. Against our expectations, we again found no significant validity effects in Experiment 2. As even task relevance could not enable subliminal faces to capture attention, we can conclude that conscious perception is a prerequisite for attention capture by fearful expressions.

In general, some “pre-attentive” discrimination of visual features related to emotional facial expressions is possible for faces presented in the periphery (e.g., Pizzagalli et al., 1999; Rigoulot et al., 2011, 2012; Calvo et al., 2014; Towler and Eimer, 2015). Yet, the peripheral presentation of our faces might have impeded effects in later occurring ERPs, as peripheral presentation of faces at both peripheral locations somehow masks the strongly delayed ipsi- vs. contralateral ERPs (see Takamiya et al., 2020; Schindler et al., 2022). Also for centrally presented well visible fearful faces (presented for 50–100 ms; unmasked), differential effects disappear at the level of the N2/EPN when attending to overlaid perceptual information (Schindler et al., 2020a; Steinweg et al., 2021) or under conditions of peripheral load (e.g., see Schindler et al., 2020b, 2021).

At face value, the conclusion that awareness might be necessary for capture of attention by peripheral fearful faces cues (or other, e.g., neutral facial expressions if task-relevant) seems to be inconsistent with some past findings (Morris et al., 1998; Dolan and Vuilleumier, 2003; Tamietto and De Gelder, 2010; Rohr et al., 2012; Rohr and Wentura, 2021). However, it should be noted that many of these studies did not control for salience differences between different masked stimuli, and when this possibility was tested, it turned out that salience was probably involved in the processing of subliminal fearful faces (Morris et al., 2002). In contrast, in the current study, we took great care to equate different stimuli in terms of their salience. In addition, studies showing assumedly subliminal processing of fearful faces are often not using the most convincing visibility tests, so that some of the processing of these stimuli could be due to residual visibility of the fearful faces (cf. Hedger et al., 2016). Finally, we presented subliminal face cues at unattended positions to test whether they capture attention. In contrast, many prior studies with masked fearful faces presented the subliminal stimuli at attended locations (e.g., Rohr et al., 2012). To the degree that directing spatial attention to a face facilitates the processing of its features (but see Finkbeiner and Palermo, 2009, for evidence to the opposite), our procedure would simply not be sensitive to these kinds of attention-dependent processing.

The present results are, thus, also in line with a view that stresses that some forms of processing facial emotional expressions of humans could require awareness (Hedger et al., 2016). We note, however, that our procedure was challenging for the participants, as we always presented two faces side by side and, thus, two relatively interesting and informative stimuli competed for our participants’ attention. It could be that the chances for the expression of awareness-independent capture of attention by subliminal fearful faces are better under less challenging conditions. Yet, we also wanted to point out that supraliminal face cues would pass the test even with the currently employed relatively high demands (cf. Khalid et al., 2017).

## Data Availability Statement

The raw data supporting the conclusions of this article will be made available by the authors, without undue reservation.

## Ethics Statement

Ethical review and approval was not required for the study on human participants in accordance with the local legislation and institutional requirements. The patients/participants provided their written informed consent to participate in this study.

## Author Contributions

MK and UA designed the experiments. TD programed the experiments. MK collected the data. DB analyzed the data. DB and UA wrote the manuscript. All authors contributed to the article and approved the submitted version.

## Conflict of Interest

The authors declare that the research was conducted in the absence of any commercial or financial relationships that could be construed as a potential conflict of interest.

## Publisher’s Note

All claims expressed in this article are solely those of the authors and do not necessarily represent those of their affiliated organizations, or those of the publisher, the editors and the reviewers. Any product that may be evaluated in this article, or claim that may be made by its manufacturer, is not guaranteed or endorsed by the publisher.
